# Fluoroquinolone heteroresistance, antimicrobial tolerance, and lethality enhancement

**DOI:** 10.3389/fcimb.2022.938032

**Published:** 2022-09-29

**Authors:** Amit Singh, Xilin Zhao, Karl Drlica

**Affiliations:** ^1^Department of Microbiology and Cell Biology, Indian Institute of Science, Bangalore, India; ^2^Centre for Infectious Disease Research, Indian Institute of Science, Bangalore, India; ^3^Public Health Research Institute and Department of Microbiology, Biochemistry and Molecular Genetics, New Jersey Medical School, Rutgers Biomedical and Health Sciences, Rutgers University, Newark, NJ, United States; ^4^State Key Laboratory of Molecular Vaccinology and Molecular Diagnostics, School of Public Health, Xiamen University, Xiamen, China

**Keywords:** antimycobacterial, oxidative stress, fluoroquinolone, respiration, N-acetyl cysteine, redox biosensor, reductive stress, resistance

## Abstract

With tuberculosis, the emergence of fluoroquinolone resistance erodes the ability of treatment to interrupt the progression of MDR-TB to XDR-TB. One way to reduce the emergence of resistance is to identify heteroresistant infections in which subpopulations of resistant mutants are likely to expand and make the infections fully resistant: treatment modification can be instituted to suppress mutant enrichment. Rapid DNA-based detection methods exploit the finding that fluoroquinolone-resistant substitutions occur largely in a few codons of DNA gyrase. A second approach for restricting the emergence of resistance involves understanding fluoroquinolone lethality through studies of antimicrobial tolerance, a condition in which bacteria fail to be killed even though their growth is blocked by lethal agents. Studies with *Escherichia coli* guide work with *Mycobacterium tuberculosis*. Lethal action, which is mechanistically distinct from blocking growth, is associated with a surge in respiration and reactive oxygen species (ROS). Mutations in carbohydrate metabolism that attenuate ROS accumulation create pan-tolerance to antimicrobials, disinfectants, and environmental stressors. These observations indicate the existence of a general death pathway with respect to stressors. *M. tuberculosis* displays a variation on the death pathway idea, as stress-induced ROS is generated by NADH-mediated reductive stress rather than by respiration. A third approach, which emerges from lethality studies, uses a small molecule, N-acetyl cysteine, to artificially increase respiration and additional ROS accumulation. That enhances moxifloxacin lethality with *M. tuberculosis* in culture, during infection of cultured macrophages, and with infection of mice. Addition of ROS stimulators to fluoroquinolone treatment of tuberculosis constitutes a new direction for suppressing the transition of MDR-TB to XDR-TB.

## 1 Introduction

### 1.1 Overview

Antimicrobial treatment of tuberculosis has led to widespread emergence of resistance, particularly to the two most effective first-line agents, rifampicin and isoniazid. The resulting disease, called multidrug-resistant tuberculosis (MDR-TB), increased in prevalence by over 20% annually between 2008 and 2016 (Lange et al., 2018). Treatment of MDR-TB requires many months with second-line agents (a fluoroquinolone and one of three injectable drugs such as kanamycin, amikacin, or capreomycin). MDR-TB that acquires resistance to second-line agents, including a fluoroquinolone, is termed extensively drug-resistant tuberculosis (XDR-TB), a disease that is exceptionally difficult to cure. Results from resistance surveys are concerning: by 2018 XDR-TB accounted for about 6% of MDR-TB cases (WHO, 2013; WHO, 2019), and the increase in XDR-TB cases was almost 10-fold between 2011 and 2018 (WHO, 2013; WHO, 2019). Finding ways to halt the progression from MDR-TB to XDR-TB is a major healthcare priority.

We are focusing on improving the effectiveness of fluoroquinolones, the most potent of the second-line drugs used against MDR-TB. Addition of a C-8 methoxy group to ciprofloxacin-like agents, as found with moxifloxacin and gatifloxacin, improves lethal action, especially against resistant mutants (Dong et al., 1998; Zhao et al., 1999). However, this structural change my be insufficient, since older, less potent quinolone derivatives are still widely used. Moreover, the fluoroquinolones are used extensively for many other infections − inadvertent pre-treatment of TB may contribute to the emergence of fluoroquinolone-resistant tuberculosis (Bernardo and Yew, 2009), especially since the quinolones generate resistant mutants (Malik et al., 2010; Malik et al., 2012a). Even when treatment is brief, pretreatment with fluoroquinolone is associated with the emergence of resistance (Ginsburg et al., 2003). Thus, the emergence of fluoroquinolone-resistant *M. tuberculosis* is likely to remain a problem until ways are developed to suppress it.

In the present review we consider three fluoroquinolone issues: heteroresistance, tolerance, and enhancement. Heteroresistant cultures contain significant subpopulations of resistant mutants but score as susceptible when tested phenotypically. Maintaining selective pressure can lead to fully resistant infections. Tolerant bacteria are not killed by antimicrobials. Studies of tolerance lead to the formulation of a stress-mediated death pathway that may be exploited. Finally, unique features of *M. tuberculosis* have led to a way to enhance moxifloxacin-mediated killing. These studies of fluoroquinolones are likely to be of broad interest, because some of the principles appear to apply to antimicrobials in general. For example, heteroresistance is a general property of bacteria, and the death pathway appears to be common to antibiotics, disinfectants, and environmental stress (Zeng et al., 2022). We begin by discussing two key characteristics of bacteria, resistance and tolerance.

### 1.2 Resistance and tolerance

Resistance occurs when an isolate has an MIC above an empirically determined breakpoint. Mechanistically, resistance is the inability of the drug to form an initial bacterial lesion and thereby the inability to block bacterial growth. In general, resistance can be caused by reduced drug uptake, drug degradation, increased efflux, or the inability of the antimicrobial to interact with its molecular target. For the quinolones, resistance arises from the failure to form drug-gyrase-DNA complexes that would otherwise rapidly block DNA replication (Drlica et al., 2019). Resistance forces the patient to rely on the immune system to clear infection. In the case of tuberculosis, immune-based clearance can be ineffective (Pawlowski et al., 2012), which makes resistance particularly problematic.

Antimicrobial tolerance is elevated bacterial survival during treatment with a lethal antibiotic in the absence of a decrease in bacteriostatic susceptibility (no increase in MIC) (Tuomanen et al., 1986). Many lines of evidence support the idea that resistance and tolerance are mechanistically distinct (reviewed in (Drlica and Zhao, 2021)). For fluoroquinolones, and likely most lethal stressors, killing arises in part from macromolecular destruction by reactive oxygen species (ROS). Tolerance appears to be specific interference with ROS-mediated effects. For example, an iron chelator and a radical scavenger reduce killing by fluoroquinolones with little or no effect on MIC (reviewed in (Drlica and Zhao, 2021)). In principle, tolerance is expected to make clearing infection difficult and contribute to tuberculosis relapse. Tolerance also contributes to elevated frequency of resistance (Levin-Reisman et al., 2017; Shee et al., 2022), probably by allowing bacterial numbers to remain high and by reducing the killing of resistant mutant subpopulations. We note that quinolones also kill bacteria by chromosome fragmentation − examples exist in which interference with ROS accumulation fails to block killing completely (Malik et al., 2007; Keren et al., 2013).

### 1.3 Mutant selection window

**Figure 1:**
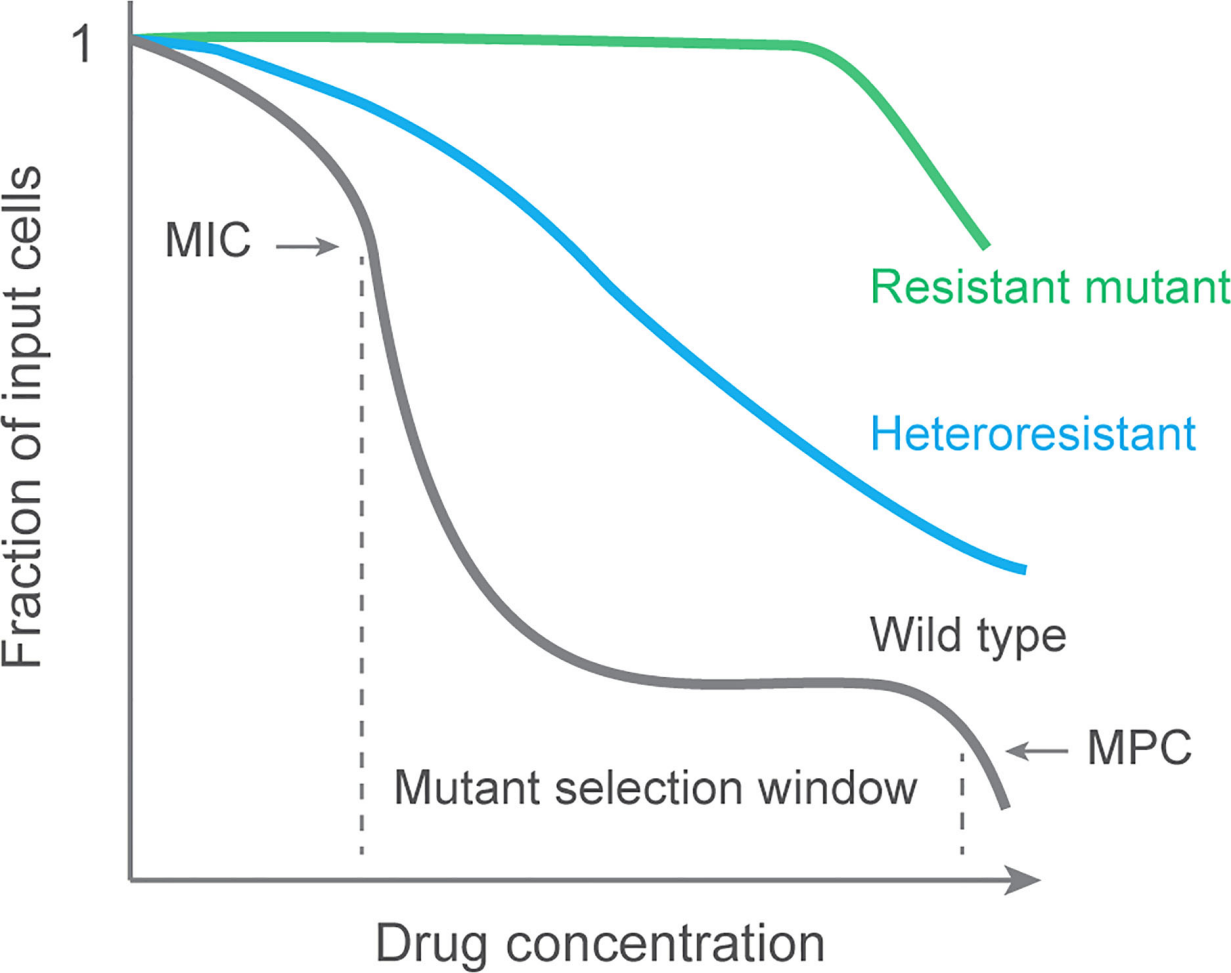
Population analysis profile and mutant selection window. Data are generated by applying a bacterial culture to a series of agar plates containing various concentrations of antimicrobial. After incubation to allow colony formation, colonies are counted, and the number is plotted for each drug concentration as a fraction of the input. Resistant cultures are unaffected by the drug until concentrations are very high. A fully susceptible culture (wild type) exhibits a sharp drop in colony recovery at MIC. A second sharp drop occurs at the MIC of the least susceptible mutant subpopulation (MPC). Selective enrichment of resistant mutants occurs at concentrations between MIC and MPC, a range called the mutant selection window (Zhao and Drlica, 2001). A population containing a mixture of susceptible and resistant subpopulations is called heteroresistant. Data for wild-type *M. tuberculosis* can be found in reference (Zhou et al., 2000).

Detection of resistant mutants at various fluoroquinolone concentrations reveals that mutants are most readily recovered when concentration exceeds wild-type MIC, which exerts selective pressure, but below the MIC of the least susceptible mutant subpopulation, a value that suppresses the outgrowth of resistant mutants. The latter value is termed the mutant prevention concentration (MPC; see **Figure 1**); The concentration range between MIC and MPC is called the mutant selection window, since mutant subpopulations are selectively amplified in that range. Validation of the selection window idea has focused on pathogens other than *M. tuberculosis* (Cui et al., 2006; Drlica and Zhao, 2007; Zhu et al., 2012; Ni et al., 2014; Zhang et al., 2014; Xiong et al., 2016), but MPC has been measured with *M*. *tuberculosis* cultures (Dong et al., 2000; Rodriguez et al., 2004) and in an animal model of tuberculosis (Almeida et al., 2007).

In principle, amplification of mutant subpopulations can be restricted by keeping relevant tissue concentrations above the MPC. However, that is difficult in practice due to the high drug concentrations required: they may have adverse effects on patients. Consequently, doses designed to cure disease tend to place drug concentrations inside the selection window, thereby selectively enriching resistant subpopulations with every treatment. Thus, designing dosing strategies to simply cure disease (Wald-Dickler et al., 2018) has a fundamental flaw with respect to the emergence of resistance.

When resistant subpopulations are detectable during infection, the overall pathogen population is heterogeneous; the infection is said to be heteroresistant. Below we discuss heteroresistance, which we consider to be an early stage in antimicrobial-mediated evolution to bacterial resistance.

## 2 Heteroresistance

### 2.1 Overview

For many bacterial pathogens, heteroresistant infections often respond favorably to antimicrobial treatment, largely because the dominant, susceptible portion of the population is controlled well enough for host defense systems to clear infection. In such situations, heteroresistance is a problem mainly for immunocompromised patients. However, a heteroresistant infection can evolve to full resistance. An example is seen with colistin resistance of *Enterobacter* (reviewed in (Band and Weiss, 2019)). An isolate was examined in which 1 to 10% of the population grew in the presence of 1000-times the colistin concentration normally used to block growth of susceptible cells. In the presence of the drug, the resistant subpopulation rapidly expanded. In this case, expansion was transient, probably reflecting induction of colistin-resistance genes. When mice were infected with the heteroresistant strain (1/10^5^ bacterial cells tested resistant), the bacteria failed to respond to a colistin treatment that protected mice infected by a fully susceptible strain. Thus, massive enrichment can occur during drug exposure.

Heteroresistance is observed with *M. tuberculosis* in both HIV-positive and HIV-negative patients (Zetola et al., 2014). Moreover, it is detected for many antimicrobials, including ethambutol, isoniazid, rifampicin, fluoroquinolones, streptomycin, pyrazinamide, and amikacin. Thus, heteroresistance is a general phenomenon with *M. tuberculosis*. It is also a general problem, because the disease is usually treated intensely for many months. During long incubations, resistant bacterial subpopulations tend to be enriched. Indeed, a quarter of MDR *M. tuberculosis* isolates can be heteroresistant to fluoroquinolones (Zhang et al., 2012; Eilertson et al., 2014). If phenotypic heteroresistance is greater than 1% by drug susceptibility testing, the infection is considered resistant (Canetti et al., 1963). Thus, with *M. tuberculosis*, heteroresistance is taken as a strong warning of future resistance. That makes rapid detection methods important.

### 2.2 Detection of heteroresistance: General considerations

For rapidly growing bacteria, heteroresistance is easily detected by observing colonies in the zone of inhibition created by a spot of antimicrobial on agar where a lawn of bacteria form (see example with E-test strips in (Pournaras et al., 2005)). If colonies inside the inhibition zone are positive for resistance using MIC-based tests, the overall population is considered heteroresistant. When those colonies continue to appear resistant after multiple rounds of growth using drug-free medium, heteroresistance is considered stable. However, many examples have been reported in which heteroresistance is lost during subculturing in drug-free medium. Such situations are termed unstable heteroresistance. The “colonies-within-the-inhibition-zone” test can be used by diagnostic laboratories to detect heteroresistance with samples that would otherwise be considered susceptible. Unfortunately, slow pathogen growth renders this method of little utility with *M. tuberculosis*.

The gold standard for demonstrating heteroresistance is finding “resistant” subpopulations in a population analysis profile (PAP (deLencastre et al., 1991); see **Figure 1**). A fully susceptible isolate will show a sharp decrease in colony number when drug concentration in agar reaches the MIC. Such is seen with laboratory isolates of *M. tuberculosis*: resistant colonies can be recovered, but they are rare (Zhou et al., 2000). A heteroresistant population is seen as a more gradual drop in colony recovery (**Figure 1**). Integration of heteroresistance data and normalization to a reference strain lacking detectible heteroresistance generates a single number to compare heteroresistance among pathogen samples.

Since performing a full population analysis is labor intensive, a variation is applied to *M. tuberculosis*. An infection is deemed resistant if the proportion of colonies that are resistant exceeds 1%. This phenotypic method can be very sensitive for mutant detection, but it has two drawbacks. First, incubation times are long due to the slow growth of *M. tuberculosis*. For example, detection of resistance to fluoroquinolones and second-line injectables by conventional methods (a two-step process) takes approximately 15–30 days. Second, subculturing from sputum samples can alter the size of the mutant subpopulation due to selective advantage or disadvantage (Metcalfe et al., 2017). Moreover, long incubation times can make the induction of resistance an important factor. For quinolones, colony number increases dramatically on agar plates over the course of days with rapidly growing bacteria (Malik et al., 2010) and over the course of several weeks with *M. tuberculosis* (Malik et al., 2012a). DNA-based detection methods have been developed to overcome these problems. For example, the time required to detect resistance by a commercial line-probe test (MTBDR*sl*) is 1–2 days (Ajbani et al., 2012). The key for detecting DNA-based fluoroquinolone resistance is knowing which nucleotide sequence changes cause resistance.

### 2.3 Detecting heteroresistance: Fluoroquinolone-resistance alleles

Most clinically relevant fluoroquinolone resistance derives from amino acid substitutions in the target protein, DNA gyrase (*M. tuberculosis* lacks the related enzyme, topoisomerase IV, which would otherwise contribute to resistance). The resistance alterations map in narrow regions of the two subunits of gyrase, GyrA and GyrB (these short regions have been termed quinolone-resistance-determining regions; QRDRs).

For GyrA, the QRDR, initially found with *E. coli* (Yoshida et al., 1990), comprises codons 90, 91, and 94 in *M. tuberculosis*. Changes at these positions probably interfere with the interaction of the carboxy end of the quinolone with the QRDR of GyrA (Aldred et al., 2014). Since the structure of this end of quinolones is common to the class, the GyrA substitutions are likely to confer resistance to all quinolones. However, different substitutions at a given codon confer different levels of protection, as indicated by different proportions of mutant recovery at different fluoroquinolone concentrations on agar plates (Zhou et al., 2000), from infected mice (Bernard et al., 2016), and among clinical isolates (**Table 1**).

**Table 1:** Examples of GyrA alleles associated with resistance *in vivo.*

G88C/A	D89N/G	A90V	S91P	D94H	D94A/Y/N	D94G	Ref
		24	6		11	42	(Ajbani et al., 2012)
		9			12	41	(Hillemann et al., 2009)
3		40	16	3	6	30	(Chakravorty et al., 2011)
		30			9	44	(Zhang et al., 2012)
	13	5	5	7	11	15	(Bernard et al., 2016)^a^

a Murine infection.

Percent of single alleles recovered from infections.

Studies with *E. coli* reveal that an A67S substitution also reduces susceptibility (Malik et al., 2006), although the main effect of this allele is on lethal action. We speculated that this substitution weakens the GyrA-GyrA interface, thereby stimulating gyrase subunit dissociation and chromosome fragmentation (Malik et al., 2006). Since the corresponding allele (A74S in *M. tuberculosis*) can be detected with *M. smegmatis* and *M. tuberculosis* in “low-level resistant” mutants, the A to S substitution also likely affects bacteriostatic activity (Zhou et al., 2000). Indeed, introduction of the allele into a laboratory strain of *M. tuberculosis* increased MIC by 2- to 4-fold (Malik et al., 2012b). Moreover, examination of purified, recombinant A74S gyrase shows a decrease in sensitivity to ofloxacin and moxifloxacin (about 8- and 14-fold reduction, respectively (Lau et al., 2011)). Thus, including the A74S allele in DNA-based tests for heteroresistance may be appropriate.

Studies with *E. coli* also associate GyrB substitutions with resistance to some quinolones (Yoshida et al., 1990). With *M. tuberculosis*, 15% of resistant isolates contain point mutations in *gyrB* (85% map in *gyrA*). These *gyrB* alleles are likely responsible for resistance, since they reduce fluoroquinolone sensitivity when present in purified, recombinant gyrases (Aubry et al., 2006; Kim et al., 2011). To define the GyrB QRDR, 19 *gyrB* alleles were transduced into a laboratory strain of *M. tuberculosis* that was then examined for susceptibility to fluoroquinolones using the phenotypic proportion method to define resistance (Malik et al., 2012b). By this test, the QRDR is almost 90 codons long.

The protective activity of gyrase mutations observed *in vitro* does not always carry over to clinical resistance. For example, the *E. coli* GyrA G81C substitution is very protective (Mustaev et al., 2014), and the equivalent substitution in *M. tuberculosis* (G88C) is readily selected on drug-containing agar (Zhou et al., 2000). But the G88C allele is rarely recovered among clinical isolates (Chakravorty et al., 2011). Conversely, not every amino acid change seen in resistant cells reduces susceptibility. Indeed, some substitutions increase susceptibility (Aubry et al., 2006). Thus, fitness is likely to play a role in determining which alleles are relevant for DNA-based assays. Nevertheless, the correlation between gyrase alleles and resistance has been good enough to encourage the development of DNA-based assays that shorten assay time for MDR-TB to XDR-TB conversion from weeks to a day. These assays tend to focus on GyrA substitutions.

### 2.4 Assays for heteroresistance

#### 2.4.1 Line probe assay

Knowledge of the GyrA QRDR allows specific amplification methods to produce DNA fragments that are characteristic of particular alleles. The fragments can then be separated by gel electrophoresis; heteroresistance is observed by the presence of both wild-type and mutant fragments (Rinder et al., 2001). Sensitivity is improved by reverse hybridization. Paper strips are prepared in which regions of wild-type DNA and mutant DNA are placed at specific spots. Regions of sample DNA are amplified by PCR, labeled, and hybridized to DNA on the strip. Resistance is scored by hybridization to mutant fragments and by the absence of the equivalent wild-type allele; heteroresistance produces a mixed result.

Commercial assay kits are available for performing line probe assays. One called MTBDR*sl* is designed to detect resistance to fluoroquinolones (GyrA alleles) and second-line injectable drugs in samples from MDR-TB cases (Hillemann et al., 2009). For fluoroquinolone resistance using *gyrA* alleles, the concordance between the phenotypic test and the positive MTBDRs*l* assay is 90% (Hillemann et al., 2009; Ajbani et al., 2012). Thus, a rapid test for fully resistant and fully susceptible cultures is in place even without including GyrB-mediated resistance.

Occasionally discordance is observed between drug-susceptibility and DNA-based tests: DNA assays indicate resistance, but only susceptibility is seen following bacterial outgrowth. This result is explained by heteroresistance, which is clear when specimens display both mutant and wild-type bands in line-probe assays (Hillemann et al., 2009). A fitness advantage would allow susceptible bacteria to dominate during the outgrowth needed for phenotypic drug susceptibility testing. That would make DNA methods more efficient at detecting resistant mutant subpopulations when applied to primary specimens. This increased efficiency might then allow the standard for resistance *via* drug susceptibility testing to be relaxed from 1% heteroresistance to perhaps 19% with DNA-based methods (Vargas et al., 2021).

Heteroresistance below the threshold, whether 1% or 19%, may not assure the emergence of resistance, but it would serve as an early warning and could affect treatment decisions. In this scenario, the reliability of a particular assay at low levels of heteroresistance is important. For example, PCR-based diagnostic tests have a specificity problem when mutant subpopulations are small, because templates from the dominant bacterial population can create false-positive signals due to mis-priming, mis-incorporation, and mis-hybridization (DNA polymerase error frequency limits sensitivity to 0.1 to 0.2%). Another challenge for PCR-based methods arises from laboratory contamination by amplicons from previous assays. Cross-contamination using open-tube assays is estimated to be almost 4% (Warren et al., 2004; vanRie et al., 2005). Closed-tube methods would reduce laboratory contamination (Huang et al., 2011; Rice et al., 2012; Hu et al., 2014), but they require improvement in sensitivity for heteroresistant infections. Thus, interpretation of DNA-based test results is likely to depend on the method employed.

#### 2.4.2 DNA sequence determination

When DNA samples are amplified by PCR and nucleotide sequences are determined for the regions of interest, results are obtained rapidly. Many laboratories have access to the Sanger sequencing method, making it a popular assay. However, sensitivity is a problem, since in some cases the mutant frequency needs to be above 50% for detection (Folkvardsen et al., 2013). In one example, Sanger sequencing reported only 3 samples as resistant of 9 scoring resistant by the proportion drug susceptibility method (Bernard et al., 2016).

Sensitivity is improved by performing the sequencing with very large numbers (millions) of parallel determinations (deep or next-generation sequencing). The general strategy uses reversible-terminator sequencing-by-synthesis technology to provide end-to-end sequencing and many short reads. In this method, genomic DNA is extracted from bacterial cells, enzymatically sheared into small fragments, and tagged with Illumina-specific DNA identifiers. These unique identifiers allow multiple DNA fragments to be sequenced at the same time. The short, tagged fragments of DNA are purified, samples are normalized to specific concentrations, pooled, and loaded into the sequencer. The data are then computationally analyzed. When the method is extended to the whole genome, sensitivity is below 5% for resistant mutants, perhaps as low as 0.2% (Nimmo et al., 2020). A disadvantage of deep sequencing is that data handling is cumbersome: bioinformatic improvements are needed for general utility (Operario et al., 2017).

#### 2.4.3 Sloppy molecular beacons

Molecular beacons are oligonucleotides in which a probe sequence is situated between ends that are complementary and form base pairs. One end contains a fluorophore and the other a quencher. Hybridization of the probe with its target sequence destabilizes the base pairing of the ends, separating the quencher from the fluorophore. The probe-target interaction is seen as fluorescence. Sloppy molecular beacons have unusually long probe sequences that allow hybridization to long target regions that can have considerable mismatch. The mismatches lower the melting temperature of the probe-target interaction as an indicator of different gyrase alleles (Chakravorty et al., 2011).

In one iteration, the sloppy molecular beacon assay amplified the *M. tuberculosis gyrA* QRDR using asymmetrical PCR. Then probing was with two sloppy molecular beacons that spanned the entire QRDR. By testing DNA targets corresponding to all known QRDR mutations, the Alland laboratory (Chakravorty et al., 2011) found that one or both sloppy beacons produced a melting temperature shift of at least 3.6°C for each mutation. That shift is readily detectable. The assay also identifies mixtures of wild-type and mutant DNA, with QRDR mutants identified in heteroresistant samples containing as little as 10 to 20% mutant DNA. Since fluorophores emitting different wave lengths are available, a single assay tube can report the presence of specific mutations associated with distinct changes in melting temperature for each fluorophore.

#### 2.4.4 Digital PCR

Studies in cancer biology are driving the development of DNA-based assays for heteroresistance. With digital PCR (Vogelstein and Kinsler, 1999), the sample is diluted into a set of wells in a multi-well plate so a given well has only a single molecule of DNA (only wild-type DNA is present in most wells). Amplification of DNA in the wells reveals either the presence or absence of mutant DNA. The fraction of wells scoring positive for mutant estimates the percent of the sample containing mutant DNA. The sensitivity of digital PCR is limited only by the number of wells tested.

Digital PCR has been used with *M. tuberculosis* by combining wild-type DNA with DNA carrying resistance alleles in *gyrA*, *katG, rpoB*, and *rrs*. This assay can reveal heteroresistance of 1 mutant to 1,000 wild-type cells (Pholwat et al., 2013). For such sensitivity with sputum, the samples must have more than 1,000 bacilli per ml (*M. tuberculosis* content, which varies among sputum samples, can exceed one million CFU (Yajko et al., 1995; Brindle et al., 2001; Diacon et al., 2007)).

#### 2.4.5 SuperSelective primers

Another strategy, also from cancer diagnosis, employs SuperSelective primers for real-time PCR assays (Vargas et al., 2016). In this test, a DNA primer is synthesized in which one region, the anchor, hybridizes strongly to a portion of the target DNA being probed. The anchor is separated from a detector region, called the “foot”, by a long stretch of nucleotides expected to mispair with the target, thereby forming a loop. The foot is designed to hybridize only with the mutant nucleotide sequence in the target. The resulting hybrid is then used to prime real-time PCR. The SuperSelective primer method detects multiple mutations in the same reaction tube by using fluorophores having different colors to discriminate among amplification products.

#### 2.4.6 CRISPR

This bacterial process recognizes and destroys foreign nucleic acids. The recognition aspect is applied to mutant detection by transcribing DNA samples from the pathogen and then incubating the transcripts with the Cas13a protein system plus a quenched, fluorescently labeled reporter RNA. When the target RNA is recognized by Cas13a, which is designed to occur only if the resistance mutation is present, collateral damage in the reporter RNA will occur, thereby eliminating quenching and generating a fluorescent signal. This method, called SHERLOCK (Gootenberg et al., 2017), has single-molecule sensitivity, similar to droplet digital PCR and quantitative PCR (qPCR). It also has point-of-care diagnostic features. The CRISPR system functions with *M. tuberculosis* (Rock et al., 2017).

#### 2.4.7 iPLEX gold

In this method, single-nucleotide primer extension incorporates a nucleotide having a distinctive mass modification for identification by mass spectroscopy (Bouakaze et al., 2011). The method can detect multiple resistance alleles in the same reaction mixture. In one application, a reconstruction experiment reported one amikacin-resistant cell per 200 wild-type cells (Zhang et al., 2013).

#### 2.4.8 Conclusions

Detection methods vary significantly in their ability to detect heteroresistance when the resistant allele is rare. They also differ in the ease of use: commercial kits are available for the line probe assays, while deep sequencing requires bioinformatics expertise. Still unknown is the clinical significance of low-level heteroresistance: not every mutant amplifies to full resistance in patients. One of the results of DNA-based assays is the realization that two general types of heteroresistance occur in tuberculosis.

### 2.5 Two forms of heteroresistance

#### 2.5.1 Mixed infections

Heterogeneity can arise from co-infection with multiple, dissimilar infecting strains of *M. tuberculosis*. These mixed infections may be common when the spread of disease leads to super-infection. High levels of mixed infection indicate poor infection control (failure to isolate patients, control of hospital air flow, etc.). They tend to occur where tuberculosis and resistant disease are common.

Mixed infections have been identified using methods that reveal very different DNA fingerprints (IS*6110* RFLP or VNTR patterns) (Shamputa et al., 2004; Kargarpour Kamakoli et al., 2017). In a report from Tashkent, Uzbekistan (Hofmann-Thiel et al., 2009), sputum samples subjected to DNA analysis showed that five of seven heteroresistant isolates were composed of different strains. Three of these mixed infections were newly diagnosed in untreated patients; consequently, continuous antimicrobial pressure is not required to create mixed infections.

#### 2.5.2 Clonal heteroresistance

Heteroresistance can also evolve along clonal lines (within-host heteroresistance). This phenomenon is common when super-infection is rare and treatment of individual patients is poor. In this scenario, intermittent drug exposure, due to suboptimal dosing and/or factors that affect compliance, allows cycles of bacterial population expansion followed by selective reduction. Spontaneous heterogeneity is expected, because the bacterial burden can be high: some tuberculosis patients harbor on the order of 10^9^ bacilli (Canetti, 1965; Mitchison, 1984). Bacterial load is probably an important factor in the emergence of resistance, since an abnormally high mutation rate does not seem to be the cause (for cultured *M. tuberculosis*, mutation rate is similar to that of other bacteria (McGrath et al., 2014)).

The complex dynamics of clonal heteroresistance are illustrated by a South African study (Post et al., 2004). The study subjects suffered from MDR-TB that persisted despite treatment for more than a year. Since the community prevalence of MDR-TB was low (0.3% in new patients, 1.7% in previously treated patients), clonal heterogeneity was more likely than mixed infection. Indeed, examination of sputum samples from 13 HIV-negative MDR-TB patients, taken at two-week intervals, showed that all contained *M. tuberculosis* having a single IS*6110* RFLP type and spoligotype pattern: superinfection was not observed.

Nucleotide sequence analysis for several genes showed that resistance patterns for infections changed during the course of sampling. For example, one patient was tested for mutations in *gyrA, embB*, and *katG* over 56 weeks of therapy. At the start of sampling, the three genes were wild type, while at weeks 4 and 6, the *katG* marker was resistant. It later returned to wild type. The *embB* marker became resistant by week 6 and remained resistant throughout the observation period. The *gyrA* gene showed a mixture of alleles at week 6; in later samples, transient changes occurred among several *gyrA* resistance forms, often mixed with wild-type alleles. After 48 weeks, *gyrA* was a mixture of resistant and wild-type alleles. By week 52, a different *gyrA* allele (D94G) became dominant. Isolates from two other patients also contained different alleles of drug-resistance genes. These marker fluctuations illustrate the dynamic and varied nature of clonal heteroresistance with *M. tuberculosis*.

The heteroresistance detected in sputum samples arises in part from independent clonal evolution in distinct regions of the lung. When surgical samples of lung were examined from 3 patients following long-term therapy, DNA IS*6110* fingerprints were identical for *M. tuberculosis* from different lung regions: the isolates within individual patients appeared to be clonally related (Kaplan et al., 2003). In one patient, a streptomycin-resistant strain was found in an open lesion, but wild-type cells were seen in a closed granuloma. Wild-type cells were also recovered from sputum. A second patient carried bacteria with two different *gyrA* resistance alleles when obtained from open lesions, while wild-type *gyrA* was recovered from two closed lesions. A third patient harbored three types of *M. tuberculosis*: 1) bacteria from apparently normal lung tissue had wild-type genes *for embB, katG*, and *rrs*, 2) cells from sputum and four pathological sites had *embB* and *katG* resistance markers but wild-type *rrs*, and 3) bacteria from another pathological site exhibited resistance for all three genes. These findings, plus similar observations in another study (Vadwai et al., 2011) and in autopsies (Lieberman et al., 2016), lead to the idea that resistance evolution occurs independently in different lung compartments and that wild-type cells can survive treatment (they may be tolerant; see discussion of tolerance below). The results of sputum analyses probably reflect granulomas from different regions opening and releasing bacteria at different times.

The complex evolution of resistance alleles arising in different lung compartments suggests that analysis of multiple sputum samples may be necessary to accurately assess the diversity of bacterial populations in an infection. Survival of wild-type cells is particularly worrisome if those cells are genetically tolerant. As indicated by *E. coli* studies, such cells would not be killed by any antimicrobial.

## 3 Antimicrobial tolerance

Antimicrobial tolerance is the ability of a bacterium to survive lethal treatment without exhibiting an increase in MIC, a measure of susceptibility to antimicrobial-mediated growth inhibition. Knowledge of how antimicrobials kill bacteria is expected to lead to methods for measuring the prevalence of tolerance (MIC-based assays are uninformative). That knowledge should also lead to strategies for restricting the selection of tolerant mutants. A key idea, based in part on quinolone studies, is that lethal stress elicits a general stress response in which ROS accumulates and damages macromolecules (reviewed in (Drlica and Zhao, 2021)). Below we outline studies with *E. coli* to provide a framework, and then we address work with *M. tuberculosis* that expands the framework.

### 3.1 The *E. coli* ROS paradigm

In 2007 the Collins laboratory reported that three diverse antimicrobials stimulate the accumulation of ROS in *E*. *coli* (Kohanski et al., 2007). ROS are thought to be byproducts of respiration, and indeed lethal doses of fluoroquinolone do stimulate a burst of respiration (Dwyer et al., 2014). Subsequent work solidified the conclusion that severe stress elicits a cellular response that is self-destructive: genes that are protective at low stress levels can become destructive at high ones (Wu et al., 2011; Dorsey-Oresto et al., 2013). We have suggested that repair of topoisomerase-DNA lesions (e.g. double-stranded DNA breaks), which is a large energy-consuming process when observed in eukaryotic cells (Hoeijmakers, 2009), stimulates increased respiration (Dahan-Grobgeld et al., 1998; Lobritz et al., 2015; Brace et al., 2016; Hong et al., 2019; Drlica and Zhao, 2021). Elevated respiration generates superoxide and subsequently hydrogen peroxide. In the presence of iron, Fenton chemistry converts hydrogen peroxide to hydroxyl radical, which damages many molecule types and oxidizes deoxynucleotides that subsequently lead to lethal, incomplete base-excision repair (Takahashi et al., 2017; Gruber and Walker, 2018; Gruber et al., 2022). That ROS cause death rather than being caused by death is indicated by the observation that ROS-mediated death continues even after removal of the primary stressor (Hong et al., 2019). Additional support for causality comes from mutations in protective genes, such as *katG* (catalase), increasing ROS-mediated death (Wang and Zhao, 2009; Dwyer et al., 2014; Luan et al., 2018).

Many aspects of Collins’ early work were challenged (Keren et al., 2013; Liu and Imlay, 2013; Imlay, 2015) as summarized in (Drlica and Zhao, 2021), which led us to seek a clear demonstration of a lethal stress response without using an experimental approach that relies on perturbing levels of ROS. We expected that the existence of a general lethal stress response would be revealed by the enrichment and characterization of anti-death mutants that were concurrently tolerant to many stressor types. Since by definition tolerance has no effect on MIC (Tuomanen et al., 1986), obtaining tolerant mutants required that we challenge bacterial cultures with an agent for which resistance (increased MIC) is selected rarely, if at all (recovery of resistant mutants would obscure the presence of tolerant mutants). After multiple rounds of screening with phenol, tolerant mutants were recovered (Zeng et al., 2022). These spontaneous, anti-death mutants of *E. coli* survived treatment by bactericidal agents that included antibiotics, disinfectants, and environmental stressors. As required, these mutants retained their bacteriostatic susceptibility (unchanged MIC) to the agents. The pan-tolerance (anti-death) phenotype demonstrated the existence of a death pathway common to many, if not all lethal stressors.

Characterization of the mutants revealed genes involved in carbohydrate metabolism (Zeng et al., 2022). In particular, mutations were found in *ptsI* (phosphotransferase) and *cyaA* (cAMP), thereby defining a novel activity of these genes as upstream regulators of the stress-mediated death pathway. The anti-death effect was reversed by genetic complementation, exogenous cAMP, or a Crp variant that bypasses cAMP binding for activation. Moreover, mutations in the same genes were obtained when screening was performed using multiple challenges with antimicrobials rather than with phenol. Downstream events that were blocked by the mutations included a metabolic shift from the TCA cycle to glycolysis and the pentose phosphate pathway, suppression of stress-mediated ATP surges, and reduced accumulation of ROS. Thus, the tolerance genes showed that upstream signals from diverse stress-mediated lesions stimulate shared, late-stage, ROS-mediated events that damage macromolecules. Cultures of these stable, pan-tolerant mutants grew normally and were therefore distinct from tolerance derived from growth defects (described below).

Phenol, alcohol, and chlorhexidine are commonly used disinfectants. Thus, pan-tolerance leads to the idea that massive, unrestricted disinfectant use could contribute to antibiotic tolerance and eventually resistance. The recent surge in disinfectant use due to the COVID-19 pandemic may provide retrospective evidence that disinfectant consumption contributes to tolerance and ultimately resistance. Particularly insidious is the possibility that pan-tolerance weakens host defenses − the lethal activity of three agents used by the immune system (hypochlorite, hydrogen peroxide, and low pH) is reduced by pan-tolerance. Since tolerance can arise as single-gene mutations, it may be more common than we realize: tolerance could threaten the widespread use of disinfectants.

A second type of tolerance derives from decreased metabolism as described in (Brauner et al., 2016). For example, it is well known that metabolic downshift, such as entering stationary phase of culture growth, interferes with quinolone lethality (Gutierrez et al., 2017). A variety of genes whose products interfere with growth, such as toxin-antitoxin pairs (HipBA, VapBC), tRNA synthetases (MetG), metabolic enzymes (PrsA, GlpD), and many other gene products that extend the lag before exponential growth following release from stationary phase (Fridman et al., 2014), have been associated with this type of tolerance. These genes are said to be part of a tolerome (Brauner et al., 2016). This down-shift tolerance is important, as it has been associated with serious clinical consequences for treatment of blood infections involving *S. aureus* (Liu et al., 2020). A unifying idea is that the basis of growth-defect tolerance is suppression of ROS accumulation by the associated metabolic downshift.

### 3.2 The *M. tuberculosis* ROS paradigm

As a first approximation, fluoroquinolones kill mycobacteria much as seen with *E. coli* and other bacteria: killing is rapid, it is partially blocked by the protein synthesis inhibitor chloramphenicol, and it is affected by the C-7 fluoroquinolone substituent (Dong et al., 1998; Malik and Drlica, 2006). As with *E. coli*, we expect ROS to play a central role in fluoroquinolone lethality with *M. tuberculosis*. Indeed, moxifloxacin increases ROS with cultured *M. tuberculosis*, as detected by an oxidation-sensitive fluorescent dye and by a redox-sensitive biosensor (Shee et al., 2022). Involvement of hydrogen peroxide is supported by a moxifloxacin-mediated increase in peroxide and the suppression of lethality by adding catalase to the growth medium (hydrogen peroxide diffuses between the cell interior and exterior, making it vulnerable to exogenous catalase-mediated degradation). Moreover, agents that interfere with ROS accumulation (bipyridyl and thiourea) lower ROS and increase survival (Shee et al., 2022). Moxifloxacin also increases the expression of genes involved in the oxidative stress response, iron-sulfur cluster biogenesis, and DNA repair (Shee et al., 2022). In addition, the idea that fluoroquinolones have two ways to kill *M. tuberculosis* is supported by ROS appearing to act at low drug concentration but killing continuing at high concentration independent of ROS (Shee et al., 2022), as observed with *E. coli* (Keren et al., 2013). Thus, in many ways fluoroquinolone action in *M. tuberculosis* is similar to that reported for *E. coli*.

Surprisingly, and in contrast with *E. coli* work, moxifloxacin suppresses oxygen consumption in *M*. *tuberculosis* and decreases expression of *M. tuberculosis* genes involved in respiration and carbon catabolism. Thus, the two bacterial species differ in the source of ROS and therefore in the early steps of the death pathway.

**Figure 2:**
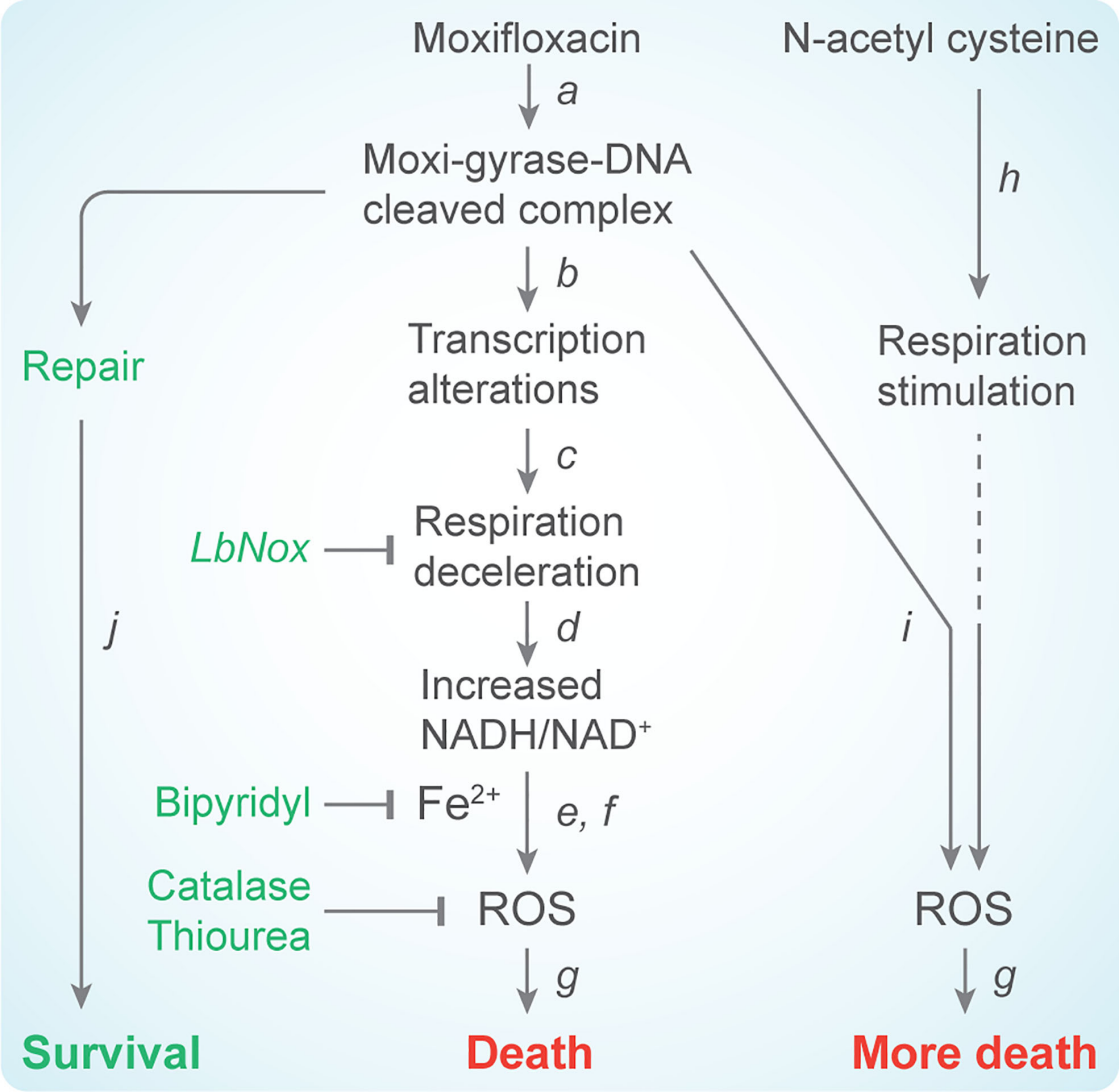
Scheme describing moxifloxacin-mediated killing of *M. tuberculosis* enhanced by NAC. **(*a*)** Moxifloxacin enters *M. tuberculosis* and traps gyrase on DNA as bacteriostatic drug-enzyme-DNA complexes in which the DNA is broken. This step is reversible. **(*b*)** The bacterium responds by down-regulating expression of genes involved in respiration. **(*c*)** The transcriptional changes result in reduced rate of respiration. **(*d*)** NADH levels and the ratio of NADH to NAD^+^ increase; over-expression of *LbNox*, an NADH oxidase, interferes with downstream events. **(*e*)** NADH increases the free Fe^2+^ pool by releasing Fe from ferritin-bound forms and keeps it in a reduced state. Bipyridyl, an Fe chelator, blocks downstream events. **(*f*)** Elevated Fe^2+^ promotes the Fenton reaction and production of hydroxyl radical. Thiourea, a radical scavenger, blocks downstream events. **(*g*)** ROS damage macromolecules and cause death in a self-amplifying process, as indicated by exogenous catalase blocking the killing when added after removal of moxifloxacin. **(*h*)** Addition of N-acetyl cysteine to cells stimulates respiration and **(*i*)** provides more ROS from moxifloxacin-mediated lesions. NAC alone does not induce ROS or trigger death. The additional ROS increase killing by moxifloxacin. **(*j*)** Repair of moxifloxacin-mediated lesions, NADH dissipation, Fe sequestration, and ROS detoxification mechanisms contribute to survival.

In *M. tuberculosis*, ROS accumulation derives from reductive stress, a phenomenon in which diminished respiration leads to the accumulation of NADH (Mavi et al., 2020). Dissipation of the NADH overload by overexpression of *Lactobacillus brevis* NADH oxidase reduces the ROS surge, diminishes free iron accumulation, and protects *M. tuberculosis* from moxifloxacin-mediated killing (Shee et al., 2022). These data fit with the known ability of NADH to mobilize bound iron and maintain iron in a reduced state (Jaeschke et al., 1992), both of which can drive the generation of hydroxyl radical *via* Fenton chemistry (Vilcheze et al., 2013). Thus, moxifloxacin-induced, ROS-mediated killing of *M. tuberculosis* appears to depend on elevated levels of NADH and iron rather than elevated respiration (**Figure 2**). Nevertheless, if respiration could be artificially increased during moxifloxacin treatment, that increase might raise ROS levels even higher and increase moxifloxacin lethality.

## 4 Lethality enhancement

Fluoroquinolones kill cells by two processes: stimulation of ROS accumulation and chromosome fragmentation. The relative contribution of the two processes to killing likely depends on quinolone structure (Malik et al., 2007), DNA repair, and quinolone concentration (Malik et al., 2007). Structural considerations favor the use of moxifloxacin (Malik and Drlica, 2006) and are not discussed further. Since ROS-mediated killing is more pronounced at low fluoroquinolone concentration, which is generally kept low to minimize adverse effects, ROS-based strategies are important. Most of our discussion of lethality enhancement with *M. tuberculosis* focuses on increasing respiration when the normal response to fluoroquinolone (moxifloxacin) exposure is to decrease it (Shee et al., 2022). Then we briefly mention suppression of repair as a way to enhance killing of *M. smegmatis* and potentially *M. tuberculosis*.

### 4.1 Cysteine reduces drug tolerance in *M. tuberculosis*

A small-molecule enhancer emerged from studies of cysteine (Vilchèze and Jacobs, 2021). When this amino acid is administered to cultured *E. coli*, its oxidation to cystine by transition metals, such as copper and iron, can mediate the production of ROS (Park and Imlay, 2003). Cysteine is also rapidly converted to cystine in *M. tuberculosis* (Vilchèze et al., 2017). As expected, the combination of cysteine and isoniazid plus rifampicin leads to cation-dependent oxidative stress and DNA damage (Vilchèze et al., 2017). The result, using cysteine at 4 mM, was a drop in *M. tuberculosis* culture density from 10^7^ CFU/ml to 0-10 CFU/ml (Vilchèze et al., 2017). In the absence of cysteine, isoniazid plus rifampicin lowered culture density by only 3 logs, and resistant bacteria emerged 7 days after treatment initiation. The effect of cysteine was not observed under anaerobic conditions or upon treatment with the iron chelator deferoxamine (Vilchèze et al., 2017), which together suggest an ROS-based phenomenon. Although cysteine fails to perturb the NADH/NAD^+^ balance expected from elevated H_2_O_2_ concentrations (Vilchèze et al., 2017), it does raise respiration, apparently by transiently shifting the ratio of bacterial menaquinol-9 (MKH2) to menaquinone-9 (MK) towards MKH2 (Vilchèze et al., 2017). That shift would prevent the entry of the bacillus into a stress-mediated, quasi dormant state that would otherwise reduce the effects of lethal stressors.

In support of the MKH2:MK hypothesis, we recently found that drug tolerance exhibited by intra-phagosomal *M. tuberculosis* depends partly on cysteine disposal mechanisms, such as Fe-S cluster biogenesis, the trans-sulfuration pathway, and mycothiol biosynthesis (Mishra et al., 2019; Mishra et al., 2021). Disruption of these processes reduces survival by ~ 9-fold upon treatment with a combination of isoniazid and rifampicin at 3X MIC (Mishra et al., 2019; Mishra et al., 2021). Cysteine alone only modestly reduces dissolved O_2_ in culture media containing *M. tuberculosis* (from ~ 75% to 60% in 300 seconds) while its combination with isoniazid more dramatically lowers dissolved O_2_ (from 80% to 40% in 300 seconds) (Vilchèze et al., 2017). Isoniazid alone does not affect dissolved O_2_ concentration in culture media (Vilchèze et al., 2017). These observations suggest that an endogenous increase in cysteine, either from inefficient fluxing or from exogenous supplementation, accelerates respiration and induces redox imbalance, thereby increasing the lethality of anti-TB drugs that act by elevating ROS. Unfortunately, cysteine, even at low, micromolar concentrations, is cytotoxic for macrophages (Vilchèze et al., 2017). However a related thiol, N-acetyl cysteine (NAC), is well tolerated by macrophages (Vilchèze et al., 2017) and patients (Nagrale et al., 2013).

### 4.2 N-acetyl cysteine during tuberculosis

Although the stimulation of lethal activity by NAC parallels that observed with cysteine, the effect on respiration is quantitatively distinct. Addition of NAC results in only a small, 0.95- to 1.25-fold increase in oxygen consumption as compared to a 4- to 5-fold increase with cysteine (Vilchèze and Jacobs, 2021). Moreover, an *in vivo* labelling study with mice indicates that NAC uptake and deacetylation may not be adequate to maintain the cellular pool of cysteine and the downstream production of glutathione, an antioxidant that would protect from stress-mediated lethality. Instead of being an active source of cysteine, NAC is readily desulfurated to produce hydrogen sulfide (H_2_S) (Ezeriņa et al., 2018). H_2_S is a signaling molecule known to increase oxygen consumption in *M. tuberculosis* by activating the energy-inefficient cytochrome BD oxidase mode of respiration (Kunota et al., 2021). It is possible that differences in the way by which cysteine (increased MKH2/MKH ratio) and NAC (cytochrome BD oxidase) stimulate respiration have distinct effects on the kinetics of oxygen consumption and thereby on the stimulation of killing associated with antimicrobial treatment of *M. tuberculosis*.

NAC also exerts host anti-mycobacterial properties by 1) increasing the production of the cytokines interleukin-2 (IL-2), interleukin-12 (IL-12), and interferon-gamma (IFN-γ), 2) decreasing other interleukins (IL-10, IL-1, IL-6) and tumor necrosis factor alpha (TNF-α), and 3) elevating host glutathione and S-nitrosoglutathione levels (Venketaraman et al., 2006; Guerra et al., 2011; Teskey et al., 2018; Cao et al., 2018). The net effect is improved immunological activities of natural killer cells and macrophages (Morris et al., 2013; Allen et al., 2015). Thus, it is not surprising that NAC displays beneficial effects in animal models of experimental tuberculosis (Palanisamy et al., 2011; Amaral et al., 2016), although significant host-species differences are seen. For example, with infected mice, NAC alone significantly reduces the bacterial load in lungs after seven days of treatment (Amaral et al., 2016), but in a guinea pig model, even 60 days of NAC treatment fails to reduce the lung bacillary load despite a decrease in lesion burden and extent of necrosis (Palanisamy et al., 2011). However, NAC does reduce the bacterial load in guinea pig spleens after 30 days of treatment (Palanisamy et al., 2011). NAC appears to delay the dissemination of *M. tuberculosis* to the spleen, perhaps due to a protective effect of NAC on lung vasculature and reduction of lesion necrosis (both lesion necrosis and loss of vascular integrity are important for extra-pulmonary dissemination of *M*. *tuberculosis*) (Palanisamy et al., 2011).

Clinical effects of NAC have been encouraging. For example, in a double-blind, randomized trial with 67 therapy-naïve TB patients, NAC, when combined with first-line anti-TB therapy (ATT), increased smear conversion from 58% to almost 96% after three weeks of treatment (Mahakalkar et al., 2017). Radiological improvement of the infected lung was evident in the NAC + ATT group (Mahakalkar et al., 2017). In another example, NAC reduced hepatotoxicity, which occurs in ~25% of patients with uncomplicated TB (hepatotoxicity can affect therapy adherence (Possuelo et al., 2008; Baniasadi et al., 2010)). Since HIV infection is one of the predisposing factors for hepatotoxicity, NAC is being tested for safety when combined with first-line ATT in patients coinfected with HIV and *M. tuberculosis*. In one study, the safety profile of the combination was similar to that of ATT alone (Safe et al., 2020). These results have encouraged an ongoing cohort study (TB-SEQUEL; ClinicalTrials.gov Identifier: NCT03702738) using a higher dose of NAC (1200 mg rather than 600 mg) to evaluate safety and smear conversion in patients with TB and TB-HIV. Since NAC appears to be useful during treatment of TB, we examined the effects of NAC on moxifloxacin-mediated killing of *M. tuberculosis*.

### 4.3 NAC stimulates moxifloxacin-mediated killing with cultured *M. tuberculosis*

NAC alone induces a rapid increase in oxygen consumption rate (Vilchèze and Jacobs, 2021; Shee et al., 2022) that completely exhausts the reserve respiratory capacity of *M. tuberculosis* (Shee et al., 2022). Adding NAC to moxifloxacin treatment reverses the respiratory slowdown seen for moxifloxacin alone, as indicated by increased oxygen consumption rate (Shee et al., 2022). Moreover, NAC-stimulated respiration enhances ROS accumulation more than seen with moxifloxacin alone. Indeed, supplementation of moxifloxacin at 1X and 5X MIC with 1 mM of NAC reduced bacterial survival by 21- and 11-fold, respectively (Shee et al., 2022). These observations are summarized schematically in **Figure 2**.

We emphasize that NAC concentrations that increase lethality have no effect on moxifloxacin MIC (Shee et al., 2022). Thus, the effect of NAC on moxifloxacin lethality is largely due to accelerated respiration and the associated ROS surge rather than a modification of the primary interaction between quinolone and DNA gyrase (cleaved-complex formation). This result strongly supports our contention that blocking growth and killing cells are mechanistically distinct. Nevertheless, NAC reduced MPC, a bacteriostatic parameter, by two-fold. Apparently killing mutant subpopulations is important in MPC determination (Cui et al., 2006).

### 4.4 NAC potentiates moxifloxacin efficacy in infected macrophages and mice

Since NAC augments host-cell glutathione biosynthesis and reduces host-generated ROS, it was unclear how NAC would affect killing of *M. tuberculosis* by moxifloxacin inside macrophages. Using an *M. tuberculosis* H37Rv strain that expresses the redox biosensor Mrx1-roGFP2 (strain *Mtb*-roGFP2), we found that moxifloxacin treatment of THP-1 macrophages, infected with *Mtb*-roGFP2, oxidizes the biosensor. Supplementation with non-toxic concentrations of NAC (1 mM to 2 mM) increased biosensor oxidation more than moxifloxacin alone, and a combination of moxifloxacin + NAC increased the level of oxidative stress by 2-fold beyond that observed for moxifloxacin alone (Shee et al., 2022). Most important, the moxifloxacin + NAC combination decreased the bacillary burden in macrophages 5-10 times more than moxifloxacin alone.

When we performed experiments with infected mice using a short moxifloxacin treatment (10 days), the moxifloxacin + NAC combination reduced bacterial burden by 4- and 12-fold more than moxifloxacin alone for lung and spleen, respectively. NAC alone had no effect on lung and spleen bacillary load (Shee et al., 2022).

Since fluoroquinolone-containing therapies are important for halting the transition of MDR-TB to XDR, we also examined the effect of NAC on the selection of moxifloxacin-resistant mutants in mice (Shee et al., 2022). We discovered that treatment with moxifloxacin alone increased the emergence of resistant strains of *M. tuberculosis*, as expected for induction of resistance by the quinolones (Malik et al., 2010; Malik et al., 2012a). NAC supplementation reduced the recovery of moxifloxacin-resistant mutants by 8-fold (Shee et al., 2022). Thus, NAC stimulates the lethal action of moxifloxacin and reduces the emergence of resistance *in vivo*.

### 4.5 NAC-mediated potentiation of lethal action with drug combinations

Several studies solidify the potential utility of NAC by showing that the compound, when added to first-line and several second-line anti-TB drug combinations, increases killing (Vilchèze et al., 2017; Vilchèze and Jacobs, 2021). For example, co-administration of NAC with inhibitors of the electron transport chain, such as bedaquiline, clofazimine, and Q203, kills cultured *M. tuberculosis* by 2 log_10_ more than bedaquiline or clofazimine or Q203 alone (Lamprecht et al., 2016). Since many antibiotics, such as isoniazid, rifampicin, and clofazimine, induce ROS in *M. tuberculosis* as part of their lethal action (Yano et al., 2011; Bhaskar et al., 2014; Piccaro et al., 2014; Tyagi et al., 2015; Nair et al., 2019), NAC likely increases respiration and the lethal action of drug combinations (Vilchèze et al., 2017; Vilchèze and Jacobs, 2021). These observations were counter-intuitive in the case of isoniazid, a prodrug that is oxidatively activated by catalase (KatG) and shows elevated activity when in combination with superoxide generators (Tyagi et al., 2015). As an antioxidant, NAC is expected to reduce the levels of free radicals such as superoxide and H_2_O_2_; thus, the mycobactericidal activity of isoniazid is anticipated to diminish when co-administered with NAC. Here, the explanation is that NAC is a poor scavenger of oxidants, such as H_2_O_2_ and superoxide, for which it has an extremely low rate constant (0.16 M^-1^s^-1^ [H_2_O_2_] and 68 M^-1^s^-1^ [superoxide], at pH 7.4 and 37°C) (Ezeriņa et al., 2018). Therefore, it is likely that enhancement of respiration and an associated increase in ROS upon treatment with NAC potentiate the antimycobacterial activity of anti-TB drugs.

NAC reduces treatment time: when combined with two first-line (isoniazid + rifampicin) or three second-line anti-TB drugs (ofloxacin + kanamycin + ethionamide or with moxifloxacin + amikacin + clofazimine), NAC reduced the time necessary to sterilize *M. tuberculosis* cultures treated with each of the combinations from 5-10 days to only 3-7 days (Vilchèze and Jacobs, 2021). Thus, NAC appears to be useful with combination therapies, as required for control of tuberculosis. Whether the contribution of the drugs in the combination therapies is additive has not been reported.

We noticed that the influence of NAC on moxifloxacin lethality differs from its effect on isoniazid and rifampicin when cells are cultured in synthetic medium. For example, with moxifloxacin the killing effect of NAC was evident at days 1 to 2 post-treatment (Shee et al., 2022): with isoniazid and rifampicin, lethality was seen only after 6 to 7 days post-treatment (Vilchèze et al., 2017).

### 4.6 ROS-mediated lethality as a kinetic phenomenon

Several lines of evidence indicate that ROS-mediated effects accelerate death without increasing the extent of killing. This phenomenon was first noticed with *S. aureus* where interference of ROS accumulation (treatment with bipyridyl plus thiourea) delayed killing by moxifloxacin (15 x MIC) for about 60 min and elevated survival by 20-fold after 120 min without an effect on minimal bactericidal concentration (MBC), a measurement involving a long incubation time (Liu et al., 2012). With *E. coli* and *M. tuberculosis*, perturbations of ROS affect the rate of killing after removal of the stressor but not the extent (Hong et al., 2019; Shee et al., 2022). The kinetic effects of ROS also fit with the increase in hydrogen peroxide being transient (Vilchèze et al., 2017) and with NAC stimulating killing of *M. tuberculosis* in mice at short incubation times (Shee et al., 2022) but not at a longer one (Vilchèze and Jacobs, 2021).

Acceleration of killing without an increase in extent has important implications for clinical application: the optimal dosing interval must be determined. If it is very short, *i.e.* frequent dosing is required, NAC might be of limited utility in resource-poor environments where patients cannot be repeatedly treated at short intervals.

### 4.7 Suppression of repair

A different form of enhancement is seen with the DNA repair pathway termed homologous repair-recombination. This system involves formation of Holliday junctions and the Ruv resolvase (Singh, 2017). We found that the absence of the *M. smegatis* Ruv resolvase increases the bacteriostatic and bactericidal activities of moxifloxacin. Treatment of *ruvAB*-deficient cells with thiourea and 2,2-bipyridyl lowers moxifloxacin killing to wild-type levels. Thus, the absence of *ruvAB* may stimulate a lethal pathway involving ROS. The hexapeptide WRWCR, which traps the Holliday junction substrate of RuvAB, potentiates moxifloxacin-mediated lethality by ten-fold (Long et al., 2015). This observation has yet to be exploited.

## 5 Concluding remarks

The fluoroquinolones are important agents for impeding the conversion of MDR-TB to XDR-TB. Human clinical studies indicate that the early bactericidal activity of moxifloxacin is similar to that of first-line anti-TB-drugs, such as isoniazid and rifampicin (Nuermberger et al., 2004; Pletz et al., 2004; Dorman et al., 2021). Moreover, a recent human clinical trial suggests that the efficacy of a four-month treatment with a combination of rifapentine and moxifloxacin was comparable to the standard six-month regimen of isoniazid, rifampicin, ethambutol, and pyramidazide (Dorman et al., 2021). However, the clinical situation is likely complex: moxifloxacin shows poor penetration into caseous regions of tubercular granulomas in a rabbit model of experimental tuberculosis (Prideaux et al., 2015; Sarathy et al., 2019). Low, local moxifloxacin concentrations may promote the emergence of fluoroquinolone resistance (Forsman et al., 2021).

General strategies have emerged for slowing the transition from MDR-TB to XDR-TB. Implementing rapid, DNA-based tests for fluoroquinolone heteroresistance will reveal the emergence of fluoroquinolone resistance before full resistance has been reached. That would enable introduction of treatment options. The most straightforward action is to advise patients of the danger, the importance of not missing doses. Another is to discontinue use of fluoroquinolone types that are only marginally effective anti-tuberculosis agents. A third is to alter the treatment protocol so that other anti-tuberculosis agents, such as rifapentine in combination with moxifloxacin, are introduced.

A second, general approach is to increase fluoroquinolone lethality to suppress the emergence of tolerance and the probability of relapse. Addition of NAC to moxifloxacin treatment is the most promising avenue, as it makes the drug more lethal and less likely to select fluoroquinolone-resistant mutants. The current problem with NAC is the kinetic nature of ROS-mediated killing, because an appropriate dosing interval is unknown. Whether that problem can be solved with derivatives of NAC is also unknown. One approach led to testing of N-acetylcysteine amide (NACA), a derivative of NAC having higher bioavailability (Vilchèze and Jacobs, 2021). This agent failed to improve the activity of drugs in *M. tuberculosis*-infected mice beyond that observed with NAC, and a more severe lung pathology was observed with isoniazid + rifampicin + NACA treatment when compared with isoniazid + rifampicin or isoniazid + rifampicin + NAC combinations (Vilchèze and Jacobs, 2021). Nevertheless, assays are now in place to explore other derivatives of NAC.

Many fluoroquinolone-related questions remain unanswered. For example, what is the prevalence of tolerance and is it a major cause of relapse? Measuring tolerance on a large scale is labor intensive (MIC plus kill curves); thus, it is not readily implemented by clinical laboratories. A bigger question that extends beyond tuberculosis is whether our massive use of disinfectants is applying sufficient selective pressure for widespread emergence of tolerance and subsequently even more resistance.

## Author contributions

All authors listed have made a substantial, direct, and intellectual contribution to the work, and each approved it for publication.

## Funding

The authors’ work has been supported by the Wellcome Trust/DBT India Alliance Grants, IA/S/16/2/502700 (AS); Department of Biotechnology (DBT) Grants (BT/PR13522/COE/34/27/2015, BT/PR29098/Med/29/1324/2018, BT/HRD/NBA/39/07/2018-19, and BT/PR39308/DRUG/134/86/2021 (AS); the Revati and Satya Nadham Atluri Chair Professorship (AS), and the National Natural Science Foundation of China (82172316, XZ).

## Acknowledgments

We thank Bo Shopsin for critical comments on the manuscript.

## Conflict of interest

The authors declare that the research was conducted in the absence of any commercial or financial relationship that could be construed as a potential conflict of interest.

## Publisher’s note

All claims expressed in this article are solely those of the authors and do not necessarily represent those of their affiliated organizations, or those of the publisher, the editors and the reviewers. Any product that may be evaluated in this article, or claim that may be made by its manufacturer, is not guaranteed or endorsed by the publisher.
